# Congenital Anchylosis of Fingers

**Published:** 1893-11

**Authors:** J. P. Coffey

**Affiliations:** Plano, Texas


### CONGENITAL ANCHYLOSIS OF FINGERS.

PLANO, TEXAS, Oct. 25, 1893.

*Editor Texas Medical Journal:*

Samuel E. Milliken, M. D., Lecturer on Surgery in the New York Polyclinic, reports in TEXAS MEDICAL JOURNAL, Oct. 9, 1893, a case of anchylosis of the fingers (congenital), and says he has not been able to find anything similar to it on record. Therefore I report the following cases:

Mr. B. has cogenital anchylosis of the second joint of all of the fingers on each hand, anchylosis of all the toes of both feet, excepting two on each foot. He has eight children, four of them have anchylosis of fingers and toes. One married daughter who has no anchylosis, but strange to say she has a child whose fingers are anchylosed. Mr. B.'s father, grandfather and great-grandfather were stiff-fingered gentlemen. Those people, all of whom I know, have never sought relief or asked any advice to correct this deformity, but on the contrary seem to be a bit proud of belonging to the straight-fingered family.

J. P. COFFEY, M. D.

---

**Alvarenga Prize of the College of Physicians of Philadelphia.**—The College of Physicians of Philadelphia announces that the next award of the Alvarenga Prize, being the income for one year of the bequest of the late Señor Alvarenga, and amounting to about one hundred and eighty dollars, will be made on July 14, 1894, provided that an essay deemed by the Committee of Award to be worthy of the prize shall have been offered.

Essays intended for competition may be upon any subject in medicine, but cannot have been published, and must be received by the Secretary of the College on or before May 1, 1894.

Each essay must be sent without signature, but must be plainly marked with a motto and be accompanied by a sealed envelope having on its outside the motto of the paper and within it the name and address of the author.

It is a condition of competition that the successful essay or a copy of it shall remain in possession of the College; other essays will be returned upon application within three months after the reward. CHARLES W. DULLES, Secretary.

---

**Edmund Andrews, M. D., LL. D.,** Professor of Clinical Surgery, Philadelphia, in the *Journal A. M. A.*, p. 613, says "No class of men or women is totally exempt from sundry ills and occasional deaths, but we could not find a single one [i. e., man or woman?—ED.] physically wrecked by childbirth in a constitution exhausted by overstudy. We found several [i. e., men or women?—ED.] who had been delicate before marriage, and became robust after bearing children."

"Occasional deaths" is good also. We are reminded of the preacher who informed his congregation that he had been expected "to die every day last week." One of them remarked that was expecting too much of any man.

---

They had asked Dr. Sandblast, the eminent surgeon, to carve the festal fowl, and he stood over it with the carving-knife held in the first position. "The incision, you will observe, gentlemen," he began dreamily, "commences a little to the left of the median line, and—oh, excuse me Mrs. Parmalee, I thought I was in the—may I help you to a little of the femur?"—*Puck.*

